# Early interruption of exclusive breastfeeding: results from the eight-country MAL-ED study

**DOI:** 10.1186/s41043-015-0004-2

**Published:** 2015-05-01

**Authors:** Crystal L Patil, Ali Turab, Ramya Ambikapathi, Cebisa Nesamvuni, Ram Krishna Chandyo, Anuradha Bose, M Munirul Islam, AM Shamsir Ahmed, Maribel Paredes Olortegui, Milena Lima de Moraes, Laura E Caulfield, on behalf of the MAL-ED network

**Affiliations:** 1 Department of Women, Children and Family Health Science, College of Nursing, University of Illinois at Chicago, Chicago, IL USA; 2 Department of Pediatrics and Child Health, Aga Khan University, Karachi, Pakistan; 3 Fogarty International Center, National Institutes of Health, Bethesda, MD USA; 4 Department of Nutrition, School of Health Sciences, University of Venda, Thohoyandou, Limpopo Province South Africa; 5 Centre for International Health, University of Bergen, Norway and Department of Child Health and Institute of Medicine, Tribhuvan University, Kathmandu, Nepal; 6 Christian Medical College, Vellore, India; 7 Centre for Nutrition and Food Security, International Centre for Diarrhoeal Disease Research, Dhaka, Bangladesh; 8 Biomedical Investigations Unit AB PRISMA, Iquitos, Peru; 9 Department of Nutrition, State University of Ceará, Fortaleza, Ceará Brazil; 10 The Johns Hopkins Bloomberg School of Public Health, Department of International Health, 615 North Wolfe Street, W2041, Baltimore, MD USA 21205

**Keywords:** Breastfeeding, Prelacteal feeding, Colostrum, MAL-ED, Nepal, Bangladesh, Pakistan, India, Brazil, Peru, Tanzania, South Africa

## Abstract

We report the infant feeding experiences in the first month of life for 2,053 infants participating in “Malnutrition and Enteric Infections: Consequences for Child Health and Development” (MAL-ED). Eight sites (in Bangladesh, India, Nepal, Pakistan, Brazil, Peru, South Africa, Tanzania), each followed a cohort of children from birth (by day 17), collecting detailed information on infant feeding practices, diet and illness episodes. Mothers were queried twice weekly regarding health status, breastfeeding and the introduction (or no) of non-breast milk liquids and foods. Here, our goal is to describe the early infant feeding practices in the cohort and evaluate factors associated with termination of exclusive breastfeeding in the first month of life. With data from enrollment to a visit at 28-33 days of life, we characterized exclusive, predominant or partial breastfeeding (using a median of 6-9 visits per child across the sites). Only 6 of 2,053 infants were never breastfed. By one month, the prevalences of exclusive breastfeeding were < 60% in 6 of 8 sites, and of partial breastfeeding (or no) were > 20% in 6 of 8 sites. Logistic regression revealed that prelacteal feeding (given to 4-63% of infants) increased the likelihood of partial breastfeeding (Odds Ratio (OR): 1.48 (95% confidence interval (CI): 1.04, 2.10), as did the withholding of colostrum (2-16% of infants) (OR: 1.63:1.01, 2.62), and being a first-time mother (OR: 1.38:1.10, 1.75). Our results reveal diversity across these sites, but an overall trend of early transition away from exclusive breastfeeding in the first month of life. Interventions which introduce or reinforce the WHO/UNICEF Ten Steps for Successful Breastfeeding are needed in these sites to improve breastfeeding initiation, to reinforce exclusive breastfeeding and delay introduction of non-breast milk foods and/or liquids.

**Electronic supplementary material:**

The online version of this article (doi:10.1186/s41043-015-0004-2) contains supplementary material, which is available to authorized users.

## Introduction

Overwhelming clinical and epidemiological evidence supports international recommendations for initiation of breastfeeding shortly after birth, avoidance of prelacteal feeding, and exclusive breastfeeding for six months, followed by the timely introduction of safe and nutritionally adequate complementary foods to prevent malnutrition and reduce morbidity in the first five years of life [1-4]. Optimal breastfeeding and the timely introduction of appropriate complementary foods not only decreases risk of diseases, especially from gastrointestinal and respiratory infections, but also promotes health and growth and development [5]. Of the 8-10 million of child deaths that occur annually, more than 90% of these deaths take place in developing countries [1]. Many of these deaths are linked to malnutrition and, in fact, an estimated 13% are related to suboptimal infant feeding practices [6,7]. Given this evidence, it is not surprising that globally, less than 25% of children 6 months of age are exclusively breastfed and for those 4 months of age, the percentage (35%) is only slightly better [6].

The importance of breastfeeding initiation is highlighted in epidemiological studies from Ghana, India and Nepal which show a relationship between timing of initiation and risk for neonatal mortality [8-10]. In Ghana, low-risk infants were at twice the risk of neonatal death if they were not breastfed within the first 24 hours after birth. Even after controlling for potential confounding factors, late initiation was associated with a 78% increased risk for mortality in India [11]. Although the causes of breastfeeding initiation delay were not explored, other research shows that primiparity [12], labor duration [13], maternal overweight [14], and perceptions about breast milk production [15] are factors associated with delays in the initiation of breastfeeding. Child birth delivery mode is another factor related to breastfeeding success. Cesarean delivery is certainly a lifesaving intervention for a mother, fetus, or both but this surgery negatively affects breastfeeding initiation and continuation [2,16-20].

Another factor that can undermine breastfeeding and the establishment of exclusive breastfeeding is prelacteal feeding [21]. In some settings, prelacteal feeds include the ceremonial provisioning of honey or rice, whereas in others it may involve water, teas, or milks [22] which can lead to early introduction with solid or semi-solid foods [23-25]. Even in the absence of prelacteal feeding, the duration of exclusive breastfeeding is often shortened by the early introduction of other liquids, solid or semi-solid foods. For example, a large survey of a rural coastal Tanzania community showed that while the mean duration of breastfeeding was two years, the average duration of exclusive breastfeeding was only 23 days as most infants were introduced to semi-solids in the form of grain-based porridges within the first two months [26]. Highlighting the importance of the first month of life, a study conducted in Peru reported that patterns in the first month of life predicted patterning in feeding throughout infancy [27]. Although less explored from an epidemiological perspective there is evidence to suggest that maternal distress associated with economic and social hardships and food insecurity plays an important role in infant feeding decisions [28-32].

The data used for our analyses were collected as part of a prospective longitudinal birth cohort study called Malnutrition and Enteric Infections: Consequences for Child Health and Development [33]. The goal of the MAL-ED study is to examine inter-relationships between diet and enteric infections over time as these relate to the development of intestinal inflammation, malnutrition and cognition in children from birth to 24 months of age. The eight MAL-ED sites are epidemiologically diverse, low-income, and located in urban (Bangladesh, Brazil, India, Nepal), peri-urban (South Africa) and rural contexts (Tanzania, Pakistan, Peru). Throughout the manuscript the sites are referred to by the following abbreviations: BGD: Dhaka, Bangladesh [34]; BRF: Fortaleza, Brazil [35]; INV: Vellore, India [36]; NEB: Bhaktapur, Nepal [37]; PEL: Loreto, Iquitos, Peru [38]; PKN: Naushero Feroze, Pakistan [39]; SAV: Venda, South Africa [40]; TZH: Haydom, Tanzania [41]. In this paper, we report and compare infant feeding experiences in the first month of life for 2,053 infants participating in MAL-ED study.

## Methods

To ensure standardized data collection, the MAL-ED Network developed common and harmonized protocols and methods to be deployed at all sites. Protocols were approved by Institutional Review Boards at each of the participating research sites and at collaborating institutions and written informed consent was obtained for each participant at each site.

The goal for each site was to recruit, retain, and follow 200 mother-newborn dyads for 24 months; the actual enrollment varied between sites to because of site-specific estimates for retention until 24 months of age. The MAL-ED sample size was selected to test hypotheses about the influences on child growth and development in the first two years of life at both the all site (N = 1600) and individual site level.

Trained study personnel identified pregnant women through a community survey as well as through health clinics or by referral. Prior to data collection, it was decided that final eligibility would be determined based on the following inclusion criteria: 1) healthy singleton newborn (≤17 days of age); 2) mother 16 + years of age; 3) family intended to stay in the study area for the next 6-month period; 4) no other child from the same family was enrolled in the study; 5) birth weight or enrollment weight was greater than 1500 grams; 6) child did not have a diagnosable congenital disease or severe neonatal disease. Although enrollment at all sites ended February 2012, sites varied as to the date when they started recruiting subjects with the earliest beginning in November 2009. Furthermore, enrollment was staggered across sites to allow for analysis of, and control for, seasonality. In total, 2,145 children were enrolled in the study.

At the time of enrollment, a structured questionnaire answered by the mother was used to collect recall data on breastfeeding initiation, the withholding of colostrum, prelacteal feeding, as well as infant, maternal and family factors including infant gender and birth weight, maternal age, parity, education and marital status. Following standard protocols [42] newborn/infant anthropometric measures (length, weight and head circumference) were taken by trained study personnel. Due to limitations on protocol changes, we were able to re-interview women at 7 of the 8 sites to collect information on type of delivery (vaginal, Cesarean). We did not collect information on the HIV status of the mothers or their infants.

After enrollment, each household was visited two times each week to collect data about illnesses (since the last contact, up to 7 days), and infant feeding practices based on the previous day’s pattern were recorded. Caregivers were specifically queried about the infant’s consumption of the following items in the previous 24 hours: breast milk, animal milk, formula, water, tea, fruit juice, other liquids, or semi-solids. On a monthly basis, a third structured questionnaire was administered and anthropometrics and information about infant feeding, immunizations, and delivery of micronutrient supplements were recorded. For this paper, we compiled the infant feeding data from the three structured questionnaires (enrollment, surveillance visits, month 1 visit) to produce a comprehensive depiction of infant feeding in the first month of life [43]. Across the sites, the median number of assessments of infant feeding per child during the first month of life varied from 6 to 9 across the sites.

Median age at enrollment at the eight sites ranged from 3 to 11 days. Of the 2,142 infants enrolled, 2,077 remained in the study during the first month, and 65 were lost to follow up (7 died, 19 moved, 13 dropped out and 26 were missing information after the enrollment visit). Of the 7 infants who died, 3 were from PKN and 1 each from BGD, INV, SAV and TZH; 6 died from infectious causes and 1 died from a congenital condition. To create a profile of infant feeding during the first month, we considered as the final visit for the period as any visit on days 28-33. There were 23 infants who had no visit between days 28 and 33 and were excluded from further analyses. Many infants had more than one visit between days 28 and 33; we therefore selected the visit closest to day 30 to represent the final visit to close out the first month. We excluded one additional infant who was visited fewer than three times after enrollment, leaving a total sample of 2,053 in the final analyses. Infants excluded were from all sites, but 51% of those excluded were from the SAV site. When enrollment characteristics of the 89 infants excluded from analyses were compared to those included, the only difference found was that the mothers of excluded infants were more educated, and this can be explained by the fact that the women in the SAV site are more highly educated than women in any other site.

We followed standard definitions to characterize breastfeeding status [44] at enrollment and each visit. Prelacteal feeding was defined as anything fed to the newborn before they were breastfed for the first time. Colostrum referred to the first milk that is in the breast or is produced immediately after childbirth. Exclusive breastfeeding was defined as breastfeeding without the introduction of other food or liquids (not even water) over the prior 24-hour period, with the exception of drops or syrups consisting of vitamins, mineral supplements or medicine. If the infant received plain water or water-based liquids such as tea or juice, this was considered predominant breastfeeding. Full breastfeeding referred to either exclusive or predominant breastfeeding. The inclusion of other milks, formula and/or semi-solids was considered partial breastfeeding. We did not consider the giving of a prelacteal feeding to nullify exclusive breastfeeding once breastfeeding was initiated. Some infants were completely weaned from the breast during the first month; when this occurred near the end of the first month, we utilized the data collected beyond the first month for confirmation.

Data were double-entered at each site by trained personnel, and were uploaded to the Data Coordination Center (DCC) on a weekly basis. Consistency checks and data cleaning were accomplished at the sites and at the DCC. Additional consistency checks and data cleaning were performed at the DCC level and sent back for investigation or correction by the corresponding site.

Data analyses were performed using STATA Version 11.0 (StataCorp LP, College Station, TX). We examined the distributions of variables, and characterized their distributions by means and standard deviations, or frequency tables as appropriate. Distributions that were not normally distributed were characterized by median and inter-quartile range (IQR) or by other percentiles of the distribution as appropriate. Because one of the goals for the analysis was to characterize the infant feeding practices in the first month of life across the eight sites, we did not focus on between-site statistical comparisons. We utilized survival analysis with age in days as the unit of analysis to characterize the pattern of decline in exclusive breastfeeding during the first month of life. Exclusive breastfeeding was treated as a one-time failure event and consequently, the child did not contribute to the risk set subsequent to the first time they departed from the exclusive breastfeeding criteria. We conducted bivariate and multivariate logistic regression analyses to examine the factors associated with partial (or no) breastfeeding at one month of age. Infant, maternal and family characteristics were evaluated for their association with partial breastfeeding at one month of age within each site as well as across all sites in an overall logistic model, which was estimated with Generalized Estimation Equations (GEE) with terms for each site as random effect included in the model. Variables with p < 0.10 were retained in the final model.

## Results

Selected characteristics of the study infants and their mothers at enrollment are shown in Table 1. The median age of enrollment varied from 3-12 days across the eight sites. The mean weight of infants at enrollment ranged from a low of 2,760 g in BGD to a high of 3,430 g in BRF. Most sites were able to comprehensively collect birth weight data (97% or greater), but only 72% from PKN and 50% from TZH had recorded birth weights because fewer enrolled infants were born at health facilities. When recorded (data not shown), the incidence of low birth weight ranged from 5-10% in BRF, INV, NEB, PEL, SAV, and TZH, with slighter higher rates, of about 20%, for BGD and PKN. Although mean maternal age was similar across the study sites, parity was much more variable across sites. In TZH and PKN, 41% and 27% of mothers, respectively, were grand multipara, and only 13% and 23%, respectively, were primipara. In contrast, at the other sites, 32-44% of study mothers were primipara. Education varied across sites; the proportion of mothers with low education, or less than 5 years of schooling, ranged from a high of 82% in PKN to a low of only 2% in SAV.

**Table 1:** **Selected maternal and newborn characteristics** ^**1**^ **at enrollment by MAL-ED study site (n = 2053)**

**MAL-ED study sites** ^**2**^	**BGD**	**INV**	**NEB**	**PKN**	**BRF**	**PEL**	**SAV**	**TZH**
N	256	242	238	269	231	293	268	256
Male child (%)	49.6	43.8	53.8	48.3	51.5	52.9	48.5	49.6
Age (d)	3 (0, 9)	10 [3,16]	12 [5,16]	11 [2,16]	9 [3,16]	4 [2,14]	8 [2,16]	7 [3,16]
Weight (g)	2,760 (400)	2,920 (460)	3,150 (460)	2,890 (490)	3,430 (510)	3,090 (460)	3,290 (480)	3,370 (470)
Maternal age (y)	24.9 (5.1)	24.1 (3.8)	26.5 (3.7)	27.6 (6.0)	24.8 (5.5)	24.2 (6.1)	26.4 (6.9)	28.5 (6.7)
Parity (%)								
1	40.6	33.7	44.1	23.0	32.0	39.3	39.1	12.5
2 – 4	57.0	61.3	54.6	50.2	58.9	47.4	53.3	46.5
>4	2.4	5.0	1.3	26.8	9.1	13.3	7.6	41.0
Cesarean delivery (%)^3^	21.5	9.8	18.9	14.5	55.7	8.9	8.8	--
Maternal education (y)								
0 - 5	64.1	37.6	25.6	81.8	8.2	21.8	1.5	37.5
6 – 10	34.8	47.1	52.1	12.3	36.8	57.7	47.8	62.5
11 – 15	1.2	15.3	22.3	5.9	55.0	21.5	50.7	0.0

^1^Presented are mean (SD) or %, except for age at enrollment for which median (5^th^, 95^th^ percentiles) are shown.

^2^Abbreviations: *BGD* Dhaka, Bangladesh; *BRF* Fortaleza, Brazil; *INV* Vellore, India; *NEB* Bhaktapur, Nepal; *PKN* Naushero Feroze, Pakistan; *PEL* Loreto, Peru; *SAV* Venda, South Africa; *TZH* Haydom, Tanzania.

^3^Data not available for 18 participants in INV, 39 in BRF and 63 in SAV. No data available for TZH.

Characteristics of the early breastfeeding practices are shown in Table 2. At the PKN site, only 7.4% were put to the breast in the first hour, and 20.9% did not initiate until day 1 or later. At the other seven sites, rates were higher with 41-83% of infants being put to the breast within one hour of birth, and 90-98% breastfed within 24 hours of birth. In SAV, 6 (2.2%) infants never received breast milk. In BGD, BRF, NEB, PEL and SAV, less than 5% of mothers reported not giving their infant colostrum, but in TZH, PKN and INV the reported rates were higher, ranging from 8.6 to 16.4%. In PKN, 63.2% of infants were given a prelacteal feeding, substantially higher than the incidence of 12.4 to 17.7% in the other South Asian sites, and 3.7-9.2% in the remaining four MAL-ED sites.

**Table 2:** **Selected characteristics of early breastfeeding practices by MAL-ED study site**

**MAL-ED study sites** ^**1**^	BGD	INV	NEB	PKN	BRF	PEL	SAV	TZH
Initiation (%)								
Within 1 hour	60.9	59.1	40.8	7.4	46.3	73.7	59.7	83.2
1 - 24 h	33.6	36.4	49.2	71.8	51.5	23.9	35.5	14.8
1 - 3 d	5.1	4.5	9.2	18.6	1.3	1.4	2.6	1.6
4 + d	0.4	0.0	0.8	2.3	0.9	1.0	0.0	0.4
Never	0.0	0.0	0.0	0.0	0.0	0.0	2.2	0.0
Not fed colostrum (%)	1.6	9.9	3.4	16.4	1.7	2.7	4.5	8.6
Prelacteal feeding (%)	13.3	12.4	17.7	63.2	6.9	9.2	3.7	5.1
Exclusively Breastfed at 30 d (%)^2^	84.7	81.0	55.5	4.5	59.7	38.2	29.5	55.9
Fully Breastfed at 30 d (%)^2,3^	90.6	87.6	70.6	7.4	68.4	77.1	58.6	58.2
Partially Breastfed at 30 d (%)^4^	9.4	11.2	29.4	92.6	29.4	22.5	36.6	41.8
Completely weaned by 30 d (%)^5^	0.0	1.2	0.0	0.0	2.2	0.3	2.6	0.0

^1^Abbreviations: *BGD* Dhaka, Bangladesh; *BRF* Fortaleza, Brazil; *INV* Vellore, India; *NEB* Bhaktapur Nepal; *PKN* Naushero Feroze, Pakistan; *PEL* Loreto, Peru; *SAV* Venda, South Africa; *TZH* Haydom Tanzania.

^2^Based on multiple assessments through the first month of life, assessed at ~30 d.

^3^Those exclusively or predominantly breastfed, assessed at ~30 d.

^4^Those who have received formula, animal milks or semi-solids in addition to breast milk, assessed at ~30 d.

^5^Those no longer receiving breast milk (verified by subsequent reporting after ~ 30 d).

As shown, the incidence of cesarean delivery ranged from 8.8 to 55.7%. Because of their known inter-relationships, we examined the bi-variate associations amongst type of delivery, delay in initiation, prelacteal feeding and avoidance of colostrum. Cesarean delivery was significantly associated with the provision of a prelacteal feed at 5 of the 7 sites, and with the avoidance of colostrum at 2 of the 7 sites that collected type of delivery data. Cesarean delivery was also significantly associated with a delay (>1 hour after birth) in breastfeeding initiation at 4 of 7 sites, and was suggestive (p = 0.09) at 1 site.

**Figure 1:**
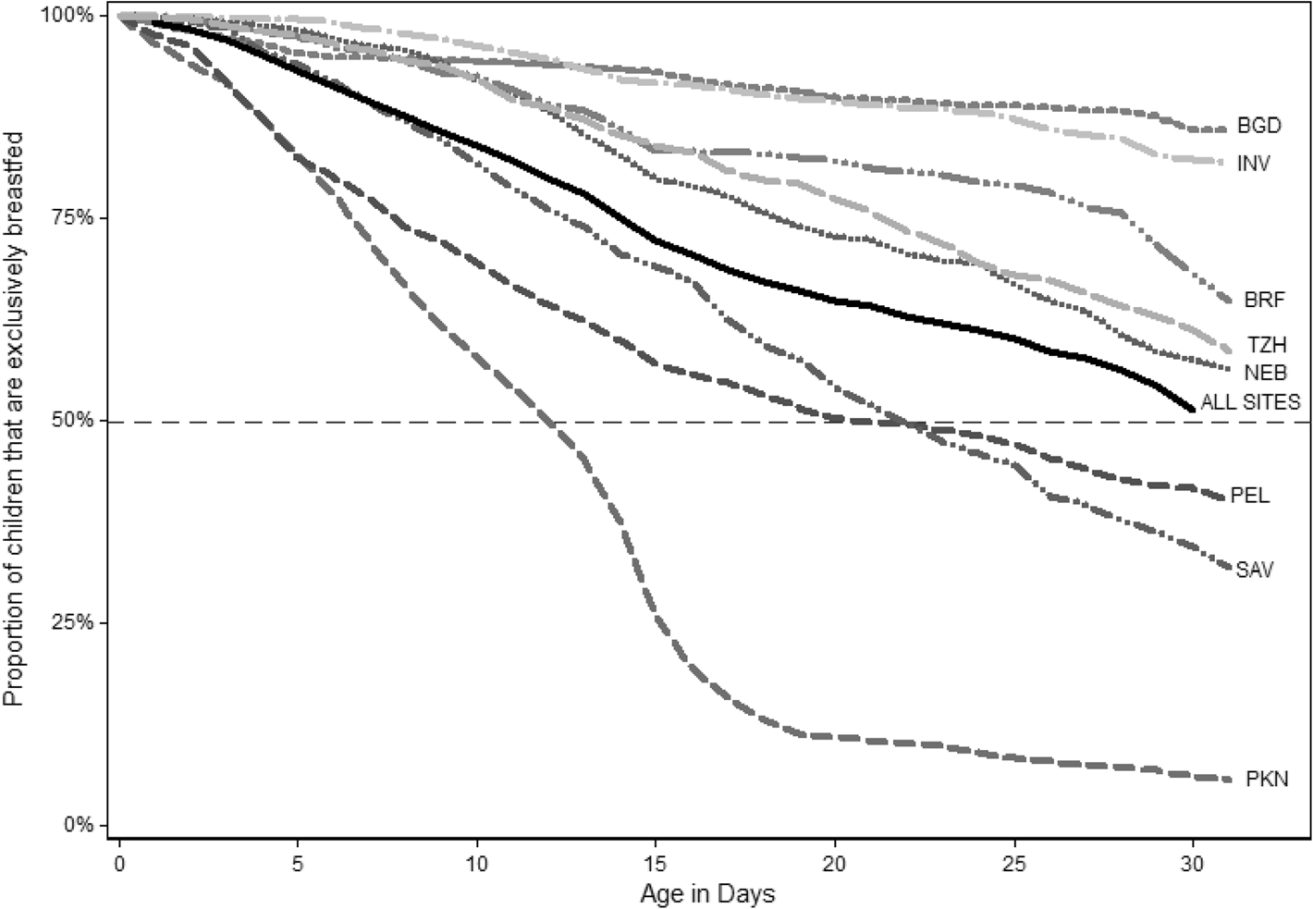
Decline in percent of newborns exclusively breastfed over time during the first month of life by MAL-ED site estimated through survival analysis with smoothing. Abbreviations: BGD: Dhaka, Bangladesh; BRF: Fortaleza, Brazil; INV: Vellore, India; NEB: Bhaktapur, Nepal; PKN: Naushero Feroze, Pakistan; PEL: Loreto, Peru; SAV: Venda, South Africa; TZH: Haydom, Tanzania.

By one month of age, the proportion of infants exclusively breastfed had declined in all sites (Figure 1 and Table 2). Only in BGD and INV were the proportions greater than 80%, with the remaining sites reporting proportions from a high of 59.7% (BRF) to a low 4.5% (PKN). As shown in Figure 1, the rate of decline in exclusive breastfeeding in PKN was steepest during the first 15 days, whereas in the other sites, the rate of decline was relatively stable across the time period although rates varied across sites. In PEL and SAV, the decline in exclusive breastfeeding was strongly related to the provisioning of water and water-based preparations to the infant; as shown in Table 2, the proportions of infants fully breastfed at day 30 was almost double the proportion of those exclusively breastfed in those sites (77.1% versus 38.2% in PEL; 58.6% versus 29.5% in SAV). The results in Table 2 also indicate that other than in INV and BGD, about one-quarter or more of the infants were partially breastfed by the end of the first month, meaning that they had already received semi-solids, formula or animal milks. Some infants were completely weaned during the first month in PEL (0.3%), INV (1.26%), BRF (2.2%) and SAV (2.6%).

**Figure 2:**
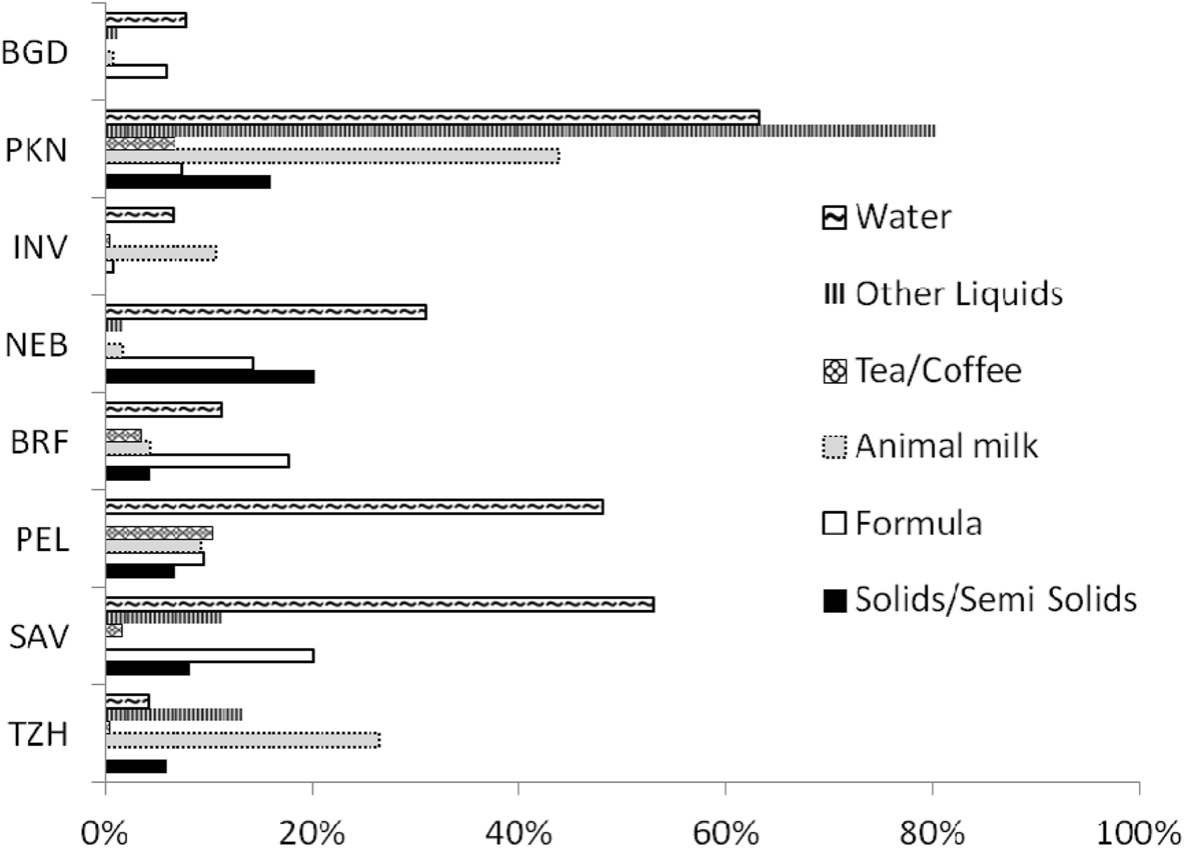
Semi-solids and liquids ever fed during the first month of life by MAL-ED site.Abbreviations: BGD: Dhaka, Bangladesh; BRF: Fortaleza, Brazil; INV: Vellore, India, NEB:, Bhaktapur, Nepal; PKN: Naushero Feroze, Pakistan; PEL: Loreto, Peru; SAV: Venda, South Africa; TZH: Haydom, Tanzania.

The proportions of infants fed plain water, different types of liquids or semi-solid foods during the first one month of life are presented in Figure 2. Formula was reportedly given to some infants in seven of the eight sites, with the highest proportions reported in SAV (20%), BRF (19%) and NEB (15%). Animal milks were also given to some infants in all sites, except for the SAV site where this is not part of infant feeding practices. Coffee was given to infants in PEL and BRF, and tea was given to infants in PEL, PKN and INV. Teas prepared in PKN and INV usually included animal milks whereas teas prepared in PEL were mostly herbal varieties without the addition of animal milks. Semi-solid porridges were given to infants in TZH, SAV and to a lesser extent in PKN. Some sites reported a high proportion of infants receiving other liquids; the identity of the other liquids varied by site and consisted of gripe or sugar water in a few sites and animal milk-based preparations (other than tea) in PKN. Additional analyses of the frequency with which these liquids or foods were fed (among those reported receiving them) indicated that except for BGD, these food items were incorporated into the feeding pattern for the child. For example, when plain water was given, the median frequency of provision in subsequent visits was 33-80% of visits during the first month in INV, NEB, PKN, PEL and SAV. The median frequency of reporting of formula (when initiated) was 43-87% across 5 sites, and for other animal milks the median frequency was 14-63% across 7 sites. Among those receiving semi-solids, the median frequency of consumption thereafter was 14-63% of visits.

The likelihood of partial (or no) breastfeeding at one month was associated with early feeding practices (Table 3). The provisioning of a prelacteal feed increased the risk of partial breastfeeding (Odds Ratio (OR): 1.48 (95% Confidence Interval (CI): 1.04, 2.10), as did the withholding of colostrum (1.63: 1.01, 2.62). There was a tendency for the initiation of breastfeeding within the first hour to be associated (p = 0.09) with decreased risk of partial breastfeeding at one month (0.81: 0.64, 1.03). Factors such as having the husband as head of household, maternal age and education were not associated with risk of partial breastfeeding, but being a first-time mother was indicative of higher risk of partial breastfeeding (1.38: 1.10, 1.75). Infant gender did not affect the likelihood of partial breastfeeding at one month. Being born of low birth weight (<2500 g) did not significantly affect risk, when evaluated in the sub-sample of infants with known birth weights (n = 1519). Type of delivery was also not significantly associated with risk of partial breastfeeding after adjusting for pre-lacteal feeding, withholding of colostrum, etc, in a model with data from 7 sites.

**Table 3:** Risk factors^1^ for partial (or no) breastfeeding at one month of life in the MAL-ED study

**Factor**	**OR (95% CI)**
Prelacteal feeding	1.48 (1.04, 2.10)
Not fed colostrum	1.63 (1.01, 2.62)
Primipara	1.38 (1.10, 1.75)
Put to the breast within 1 h	0.81 (0.64-1.03)

^1^Estimated with generalized estimation equations (GEE) with sites included as random effects. This is the reduced model including variables with p < 0.10, see text for details.

## Discussion

The WHO recommends that all infants receive breast milk beginning within an hour of their birth, that they not be given a prelacteal feeding, and that they be fed only breast milk until they reach 6 months of age. As shown here, through multiple practices including delayed initiation, the withholding of colostrum, the offering of prelacteal feeding and the provision of liquids and semi-solids, the practice of optimal breastfeeding is compromised in each of these sites. There is variability in the degree and manner through which this occurs across the sites. Overall, 40-83% of study newborns were put to the breast within an hour after birth, except in the PKN site where mothers reported that this rarely occurred. Over 60% of infants received a prelacteal feeding in PKN in contrast to less than 20% elsewhere – including the three other sites in South Asia. Most -- but not all newborns -- were given colostrum. The proportion of infants exclusively breastfed at one month varied greatly among sites, from a low of 5% to a high of 85%. In some sites, the primary reason for this decline was the provisioning of water or water-based preparations, however, the proportions of infants partially breastfed at one month (most commonly between 22-42%), indicate that significant numbers of infants are receiving animal milks, alone or in preparations, and semi-solid foods. Some are already weaned from the breast. Taken together, these data provide a compelling picture of vulnerability resulting from very early care and infant feeding decisions made for these neonates living in diverse settings.

Our analyses indicate that very early infant feeding practices, which include the provisioning of prelacteal feeds and the withholding of colostrum, affect subsequent breastfeeding patterns, specifically in this case, whether or not the infant will be given other milks or semi-solids by the end of the first month of life. Both practices are known to delay the initiation of breastfeeding, and we confirm that across these sites, they increase the likelihood of partial (or no) breastfeeding in the first month. Delayed initiation (>1 hour of birth) may be due to other factors as well, but as shown here was only marginally associated with increased risk of partial breastfeeding. Primipara, who are attempting breastfeeding for the first time, were also found to be at risk of moving to partial (or no) breastfeeding.

Less than optimal early infant feeding practices have implications for infant health and survival. Although many view the provision of water or teas to infants to be innocuous and not interfere with breast milk production, predominant breastfeeding has been shown to elevate risk of mortality in the first 6 months of life, and risks associated with partial breastfeeding are even higher [45,46]. Late initiation of breastfeeding (>24 h) has been shown in more than one study to increase risk of early cessation of exclusive breastfeeding and the risk of dying [9,15]. Prelacteal feeding exerts multiple negative consequences as well – increasing risk of cessation of exclusive breastfeeding [21], of diarrheal and respiratory illness, and of mortality [8,11,47].

The MAL-ED study sites were chosen, in part, because of on-going research on enteric infections at these sites. Thus, our site data should not be interpreted as representative of the situation in the entire country or even for a broader region; however, it is worthwhile to compare our results with previously published data from these countries. Overall, the proportions of MAL-ED infants receiving prelacteal feeds were much lower than those reported nationally or for the same region within each country [24,48-54]; clear exceptions to this were PKN and NEB. In PKN, the study frequency of prelacteal feeding of 63% is in line with the regional and national incidences of 55 and 65%, respectively [48], and in NEB, the incidence of 18% is similar to estimates of 17% from a recent study in the region [55] and 13.8% for another region of Nepal [56]. Incidences of early initiation of breastfeeding (within 1 hour of birth) in NEB, BRF SAV, TZH and INV were of comparable magnitude to regional (sub-national) or national figures [49,51,52,54,57], but were greater than regional/national figures in PEL and BGD [50,53], and lower than estimates in PKN [48]. In general, our sample estimates correspond to the statistics on early initiation of breastfeeding reported by WHO region [58].

It is difficult to compare the prevalence of exclusive breastfeeding at one month collected prospectively through multiple visits with national or regional survey based on a single interview and aggregated for infants < 2 months of age. However, in general, the prevalence of exclusive breastfeeding at one month in the MAL-ED sites are lower than those reported for the corresponding larger region or area for five of our sites (PKN, NEB, TZH, SAV, PEL) [48-51,54], but higher in the three other sites (INV, BRF, BGD) [52,53,57].

There are numerous strengths to the present study design and methodology. We used a common, harmonized protocol to collect comparable information in eight unique study sites all located in developing regions of the world. Study personnel were trained and various consistency checks and control measures enhanced data quality. Mothers were queried just after delivery and twice weekly regarding their infant feeding practices, allowing us to collect current information as feeding practices changed with time.

The main limitation of this study is that it was not designed specifically to assess risk factors associated with partial breastfeeding. This paper results from a post hoc analyses of longitudinal data collected as part of the larger MAL-ED study – a study designed to examine the influences of enteric infections (diarrhea), dietary intake, and gut function on infant growth and development. For the analyses presented here, we examined the minimal detectable differences in the odds for various risk factors. For all-site analyses, with 80% power and an alpha level of 0.05, we are able to detect differences in the odds greater than 20%, and for individual site analyses, differences of 80%-200% are detectable depending on the site and risk factor. A study designed to examine risk factors for partial breastfeeding would likely have included additional factors that were not collected as part of the MAL-ED study. However, given the variation in breastfeeding practices, we were able to identify multiple salient factors influencing partial breastfeeding in the first month across the 8 sites.

Another component of this limitation is that infants were enrolled from birth to 17 days, and thus, the period of maternal recall of initiation and the level of certainty regarding breastfeeding practices in the early days varies across the sites. However, 55% of infants were enrolled by 7 days of life, and it should be noted that the longer period of time from birth to enrollment likely makes our results paint a more optimistic picture of breastfeeding practices in these sites, because we might have missed the earliest interruptions in exclusive breastfeeding. Also, missing information on birth weight and type of delivery at some sites precluded our ability to fully investigate these risk factors. Last, we did not collect additional information at each visit that would allow us to understand the reasons for the reported feeding practices. The MAL-ED study was designed to be an observational study, and we did not want to call attention to specific practices and thereby influence the results. Ultimately, we wish to detect differences in early infant feeding practices in order to relate this information to other data on gut function, enteric illness, child growth and development, all of which are being simultaneously collected in the study.

Across these eight MAL-ED sites, breastfeeding is nearly universal, and the generally rapid initiation of breastfeeding, the low frequency with which prelacteal feeding or the withholding of colostrum are reported, all support optimal feeding and infant health. Yet, liquids were introduced frequently to infants in the first month of life and in some sites semi-solids were beginning to appear in the young infant’s diet. We show that when breastfeeding initiation is not the ideal, and when the mother is inexperienced, there is increased risk for an early move away from exclusive breastfeeding. Infant feeding practices develop as a result of multiple influences attributable to the mother-child dyad, the extended family, the broader local culture as well as from public health or health care provider messages which seek to promote health [59]. In some sites, such as in Peru, Brazil, India and Bangladesh, a relatively well developed care setting exists, whereas for sites in Nepal, South Africa, Tanzania and Pakistan little to no programmatic activity exists to promote optimal breastfeeding practices. The Baby-Friendly Hospital Initiative (BFHI) was initiated in 1990s to strengthen health facility support for the establishment of early and exclusive breastfeeding through adherence to 10 principles for optimal breastfeeding [60]. Early support/teaching to establish good technique particularly for the first-time mother, no prelacteal feeding, the giving of colostrum, putting the baby to the breast within 30 minutes of delivery are important principles in that strategy. Some have expanded the framework to the community [61,62], and in 2009 WHO/UNICEF revised and expanded this initiative [63]. Our results underscore the need to implement and/or strengthen these and other proven programmatic efforts in both maternity and pediatric services to support and protect exclusive breastfeeding between birth and the likely first postnatal pediatric care visit. The short and long-term implications of the early feeding experiences reported here on enteric illnesses, and child growth and development will be the subject of additional analyses of MAL-ED data.

## Additional file

Additional file 1:
**MAL-ED Network Investigators and Institutional Affiliations.**
