# Supplementary material for: Early interruption of exclusive breastfeeding: results from the eight-country MAL-ED study
Source: J Health Popul Nutr. 2015 May 1;34:10. doi: 10.1186/s41043-015-0004-2 (PMC5025973; doi:10.1186/s41043-015-0004-2)
Supplement: Additional file 1: — MAL-ED Network Investigators and Institutional Affiliations. [file 41043_2015_4_MOESM1_ESM.doc]

Additional file 1

**MAL-ED Network Investigators and Institutional Affiliations**

Maribel Paredes Olotegui^1^

Cesar Banda Chavez^1^

Dixner Rengifo Trigoso^1^

Julian Torres Flores^1^

Angel Orbe Vasquez^1^

Silvia Rengifo Pinedo^1^

Angel Mendez Acosta^1^

Imran Ahmed^2^

Didar Alam^2^

Syed Asad Ali^2^

Zulfiqar A Bhutta^2^

Sajad Memon^2^

Shahida Qureshi^2^

Muneera Rasheed^2^

Anita KM Zaidi^2^

Sajid Soofi^2^

Ali Turab^2^

Aisha K Yousafzai^2^

Ladaporn Bodhidatta^3^

Carl J Mason^3^

Sudhir Babji^4^

Anuradha Bose^4^

Sushil John^4^

Gagandeep Kang^4^

Beena Kurien^4^

Jayaprakash Muliyil^4^

Mohan Venkata Raghava^4^

Anup Ramachandran^4^

Anuradha Rose^4^

William Pan^5,6^

Ramya Ambikapathi^6^

Danny Carreon^6^

Vivek Charu^6^

Leyfou Dabo^6^

Viyada Doan^6^

Jhanelle Graham^6^

Christel Host^6^

Stacey Knobler^6^

Dennis Lang^6^

Benjamin McCormick^6^

Monica McGrath^6^

Mark Miller^6^

Archana Mohale^6^

Gaurvika Nayyar^6^

Stephanie Psaki^6^

Zeba Rasmussen^6^

Stephanie Richard^6^

Jessica Seidman^6^

Vivian Wang^6^

Rebecca Blank^7^

Michael Gottlieb^7^

Karen H Tountas^7^

Caroline Amour^8^

Estomih Mduma^8^

Buliga Mujaga Swema^8^

Tahmeed Ahmed^9^

AM Shamsir Ahmed^9^

Fahmida Tofail ^9^

Rashidul Haque^9^

Iqbal Hossain^9^

Munirul Islam^9^

Mustafa Mahfuz^9^

Dinesh Mondol ^9^

Ram Krishna Chandyo^10^

Prakash Sunder Shrestha^10^

Rita Shrestha^10^

Manjeswori Ulak^10^

Robert Black^11^

Laura Caulfield^11^

William Checkley^11,6^

Ping Chen^11,6^

Margaret Kosek^11^

Gwenyth Lee^11^

Pablo Peñataro Yori^11^

Laura Murray-Kolb^12^

Barbara Schaefer^12,6^

Cláudia Abreu^13^

Alexandre Bindá^13^

Hilda Costa^13^

Alessandra Di Moura^13^

Jose Quirino Filho^13,6^

Álvaro Leite^13^

Aldo Lima^13^

Noélia Lima^13^

Ila Lima^13^

Bruna Maciel^13^

Milena Moraes^13^

Francisco Mota^13^

Reinaldo Oriá^13^

Josiane Quetz^13^

Alberto Soares^13^

Erling Svensen^14^

Tor Strand ^14^

Crystal L Patil^15^

Pascal Bessong^16^

Cloupas Mahopo^16^

Angelina Mapula^16^

Cebisa Nesamvuni^16^

Emanuel Nyathi^16^

Amidou Samie^16^

Leah Barrett^17^

Jean Gratz^17^

Richard Guerrant^17^

Eric Houpt^17^

William Petri^17^

Rebecca Scharf^17^

Binob Shrestha^18^

Sanjaya Kumar Shrestha^18^

**Institutions**

^1^A.B. PRISMA, Iquitos, Peru

^2^Aga Khan University, Naushahro Feroze, Pakistan

^3^Armed Forces Research Institute of Medical Sciences, Bangkok, Thailand

^4^Christian Medical College, Vellore, India

^5^Duke University, Durham, NC, USA

^6^Fogarty International Center/National Institutes of Health, Bethesda, MD, USA

^7^Foundation for the NIH, Bethesda, MD, USA

^8^Haydom Lutheran Hospital, Haydom, Tanzania

^9^icddr,b, Dhaka, Bangladesh

^10^Institute of Medicine, Tribhuvan University, Kathmandu, Nepal

^11^Johns Hopkins University, Baltimore, MD, USA

^12^Pennsylvania State University, University Park, PA, USA

^13^Universidade Federal do Ceara, Fortaleza, Brazil

^14^University of Bergen, Norway; Haydom Lutheran Hospital, Haydom, Tanzania

^15^University of Illinois at Chicago, IL, USA

^16^University of Venda, Thohoyandou, South Africa

^17^University of Virginia, Charlottesville, VA, USA

^18^Walter Reed/AFRIMS Research Unit, Kathmandu, Nepal
